# Encounters with Persons Who Frequently Use Psychiatric Emergency Services: Healthcare Professionals’ Views

**DOI:** 10.3390/ijerph17031012

**Published:** 2020-02-05

**Authors:** Manuela Schmidt, Sigrid Stjernswärd, Pernilla Garmy, Ann-Christin Janlöv

**Affiliations:** 1 Faculty of Health Science, Kristianstad University, 291 88 Kristianstad, Sweden; pernilla.garmy@hkr.se (P.G.); ann-christin.janlov@hkr.se (A.-C.J.); 2 Department of Health Sciences, Lund University, 221 00 Lund, Sweden; sigrid.stjernsward@med.lu.se

**Keywords:** caring, content analysis, emergency care, encounter, experiences, interpersonal communication, person-centeredness, mental health nursing, therapeutic relationships

## Abstract

Encounters and interactions between healthcare professionals and patients are central in healthcare services and delivery. Encountering persons who frequently use psychiatric emergency services (PES), a complex patient group in a complex context, may be particularly challenging for healthcare professionals. The aim of the study was to explore healthcare professionals’ experiences of such encounters. Data were collected via individual interviews (N = 19) and a focus group interview with healthcare professionals consisting of psychiatric nurses, assistant nurses, and physicians. The data were analyzed with qualitative content analysis. This study focused on the latent content of the interview data to gain a rich understanding of the professionals’ experiences of the encounters. Two themes were identified: “Nurturing the encounter with oneself and colleagues for continuous, professional improvement” and “Striving for a meaningful connection with the patient”. The professionals experienced their encounters with persons who frequently use PES as caring, professional, and humane processes. Prerequisites to those encounters were knowing and understanding oneself, having self-acceptance and self-compassion, and working within person-centered cultures and care environments.

## 1. Introduction

Encounters and its interaction between healthcare professionals and patients are central in mental healthcare services and delivery [1,2], and important across all patient groups and healthcare settings. However, what constitutes an encounter remains difficult to define [3,4], mainly because different context-specific attributes highlight different important factors in the interaction. Encounters grow in difficulty with the growing complexity of the people seeking care, their needs, and the context. Thus, encounters with persons who frequently use psychiatric emergency services (PES), a complex patient group in a complex context [5], may be particularly challenging for healthcare professionals. Understanding how healthcare professionals experience such encounters could contribute to increased awareness about their own attitudes and preconceptions and to improved understanding of the patients’ situation and needs.

### Background

The quality of encounters and its interactions between healthcare professionals and patients has a profound impact on healthcare outcomes, patients’ experiences of healthcare services, and patient satisfaction with care [6,7,8]. Since any encounter between a healthcare professional and a patient is characterized by power imbalance, asymmetry, and differences in expectations [3,6,9], healthcare professionals ought to be aware of how they encounter the patients.

In healthcare literature, the concept of “encounter” is referred to in different ways, such as interpersonal interactions, relationships, professional communication, meetings, or dialogues, and these are often used synonymously [3]. In the psychiatric context, the term therapeutic relationship or alliance is commonly used [10]. In the healthcare context, some view an encounter as the same as an interaction [3,11], others see it as a special human-to-human relationship [12] or interpersonal process [1], and still others focus on particular kinds of encounters, such as caring, uncaring, or meaningful encounters [2,13,14,15,16]. We chose to apply a broad definition in this study to capture as many dimensions as possible of healthcare professionals’ experiences of encounters: direct interactions and interplay between healthcare professionals and patients including diagnosing, evaluating, and treating the patient’s healthcare needs and any other kind of being and acting around the patient, including the actions, thoughts, and feelings of both involved parties. Those encounters can vary widely in duration and can include the use of information and communication technologies such as telephones, i.e., an encounter can also be faceless. Encounters also include *how* healthcare professionals encounter patients, i.e., how they behave based on their own attitudes and preconceptions.

Healthcare professionals identified “listening”, “empathy”, and “understanding the subjective experience of the patients” as important interpersonal skills in an acute psychiatric healthcare setting [17] and essential to establishing meaningful and caring interactions with patients. However, the healthcare professionals who participated in that study also felt pessimistic about persons who frequently visited acute psychiatric care settings [17]. Other healthcare professionals were tired of encountering revisiting patients who suffered from mental illness [18] or described such patients as difficult, hard to treat and not benefiting from psychiatric interventions [5,19,20], which would inevitably have an impact on those patients’ care.

Persons who frequently use PES may be particularly challenging to encounter as not every encounter may be entered voluntarily by the patients. Police involvement, compulsory treatments, or violent patient behavior could complicate establishing interpersonal interactions and caring encounters. The hectic, stressful, and unpredictable nature of PES may make encountering those patients more challenging [21] and might result in superficial or shallow nursing care [14,22], focused on tasks and administration at the expense of developing interpersonal interactions and person-centered relationships [23]. A literature review of nurses’ experiences of delivering care in acute mental care settings revealed that nurses constantly had to balance competing perspectives and conflicting tasks concerning safety, risk assessment, enforcement of treatment, advocacy, and mitigating power with recovery-oriented care, autonomy, and the promotion of patients’ rights [23]. Conversely, a study of the experiences of persons with mental illness in need for acute care showed that caring experiences and understanding of their emotional vulnerability were lacking, and the patients felt judged and stigmatized by healthcare professionals [24]. In another study, patients in an acute psychiatric care unit reported receiving care and support from other patients, not healthcare professionals [25].

Caring encounters have been identified by both professionals and patients in Sweden as central need among persons who frequently use PES [26,27]. Not only are those encounters important for identifying patients’ healthcare needs [3], but they also become a goal or intervention in themselves meeting patients’ human needs such as being confirmed as a person. Providing good encounters within the healthcare system is required by Swedish legislation, local directives, and policies [28,29,30]. Good encounters are based on respect for all human beings, enhancing the patient’s dignity, autonomy, and integrity, and building a trustful and caring relationship.

Because the healthcare professionals are those responsible for establishing, initiating, and inviting patients to encounters, it is important to understand their perspectives on this situation. The aim of the study was to explore healthcare professionals’ experiences of encounters with persons who frequently use PES.

## 2. Method

This study employs a qualitative design due to the study’s exploratory nature and focus on human experiences. Hereafter, persons who frequently use PES are referred to as patients and healthcare professionals as professionals.

### 2.1. Context

Data were collected at one PES in southern Sweden, comprising a psychiatric emergency department and an acute psychiatric care unit. It covers a catchment population of about 200,000 inhabitants of both rural and urban areas. The PES operates 24 h day/7 days a week. Telephone counselling is a well-integrated and common part of the work of the PES.

### 2.2. Participants

The participants represented different professions working at the PES including assistant nurses, registered nurses with specialized education in psychiatry, and intern and resident physicians. Potential participants were informed of the study by the first author through workplace meetings, a video recorded by the first author, and an information email about the study’s aim, data collection methods, and their right to refuse participation. A purposeful sampling approach was applied to secure sample variation among participants in terms of age, gender, profession, work experience, and cultural background. In total, 21 professionals were asked to participate in the study; 2 declined, thus the final sample was 19 participants. A detailed description of the participants can be found in Appendix A.

### 2.3. Data Collection

#### 2.3.1. Individual Interviews

Data were collected in individual interviews with 19 professionals during October and November 2018. The semi-structured interview guide contained questions about the professionals’ experiences of the encounters with the patients as shown in Table 1. The individual interviews were conducted by the first author and lasted an average of 51 min (range, 27–86 min). Two pilot interviews, conducted by the first and last authors, were included in the analysis.

#### 2.3.2. Focus Group Interview

Of the 19 professionals who participated in the individual interviews, 6 were purposefully selected based on age, gender, profession, and work experience to participate in a focus group interview in May 2019. The focus group provided an important complementary data set as it allowed for deeper exploration of differences of experiences narrated during the individual interviews. The focus group guide was constructed by dividing preliminary results from the individual interviews into five domains: equal treatment for everybody, encounter adjustment, hindrances and facilitators in encounters, the role of the professional, and emotions. The focus group interview was conducted by the first and last authors and lasted 90 min. All interviews were conducted during working hours at the participants’ workplace.

*Within-method triangulation* allowed a more comprehensive picture of the results, and thus a clearer and deeper understanding of the phenomenon [31], and it increased the trustworthiness of the study [32]. All interviews were audio recorded and transcribed verbatim.

### 2.4. Analysis

The transcribed texts were analyzed with qualitative content analysis based on Graneheim and Lundman [33], which has been useful in nursing and health sciences as it emphasizes the analysis of experiences, perceptions, and attitudes. This study focused on the latent content, making it possible to understand the phenomenon through interpreting the professionals’ experiences. The analysis was carried out inductively, i.e., the themes emerged from the data and were thus text-driven [34]. The analysis followed a systematic two-stage process. Stage 1 consisted of several steps: (1) identifying relevant text passages from the individual interviews and forming them into a single text about participants’ experiences of encounters with patients, the unit of analysis; (2) reading this text several times to gain a sense of the whole; (3) dividing the text into meaning units; (4) condensing the meaning units into descriptions close to the text considering the context and the aim of the study, and then into interpretations of the condensed meaning units; (5) abstracting the interpreted meaning units into sub-themes which were compared for differences and similarities; and (6) finally formulating the preliminary themes that were used in the focus group guide. Examples of the analysis process and development of the sub-themes can be found in Appendix B.

After the focus group interview, the analysis process continued to Stage 2, analyzing the text from the focus group interview according to steps 2 to 6, which largely confirmed the preliminary results of the individual interviews. The group interactions between the focus group participants revealed consensus among them. The analysis was circular and moved back and forth between the parts and the whole of the text and between the analysis steps [33]. Initially, 20 sub-themes were abstracted through individual and joint discussions among the first, third, and fourth authors. The sub-themes were then aggregated into 11 sub-themes and 2 themes. Thereafter, the second author entered the analysis process by reflecting individually upon the preliminary results, which were then once more discussed by all authors until consensus was reached. The final results consist of 10 sub-themes and 2 themes.

The study was conducted in accordance with the Declaration of Helsinki [35]. The Regional Ethical Review Board found no obstacles to conducting the study (2018/569).

## 3. Results

The interpretation of the text revealed that professionals experienced encounters with patients, i.e., persons who frequently used PES, as situations in which they aimed to treat all patients equally, with ethical consideration, and in line with human values. The study showed that each patient was acknowledged as any other user of the healthcare system and as a fellow human being who was unique. Each encounter was seen as individual and was conducted with as much respect, kindness, humility, confirmation, and empowerment possible and was adjusted to the patients’ healthcare and human needs. The study also showed that professionals thought it was equally important to have a non-judgmental and open-minded attitude towards the patients, which allowed them to meet the patients without preconceptions. The study further revealed that professionals also attempted to reset before each encounter and not allow previous difficult encounters to influence their current and/or future encounters with this patient. This way, the professionals experienced each encounter with the patient as the first and focused on the current encounter in the here and now.

### 3.1. Structure of the Themes

Two themes emerged from the analysis process. Each theme included several sub-themes, as shown in Table 2. Quotes were chosen to exemplify the themes.

### 3.2. Nurturing the Encounter with Myself and Colleagues for Continuous, Professional Improvement

Encounters with persons who frequently use PES required highly professional behavior from the participants. The relationship with oneself and with colleagues played an essential role and was a precondition for good patient encounters and for their own learning process, level of professionalism and well-being.

**Allowing for constant learning from experience.** Professionals learnt from their numerous experiences of encounters with patients by assessing, analyzing, and sorting them into groups of similar encounters. This way they became familiar and routinized with any possible situation in patient encounters and could eventually rely on their experiences to interact intuitively and naturally with the patients.

*“And then what happens is that after a while once you’ve met—as you have the advantage of doing a lot in an emergency department—you get to meet a lot of different people and you add it all to your bank of experience, and sometimes it doesn’t always work out right and then you have to work through it and evaluate it, and then next time it will work out. So that, yeah, it’s like you build up this bank of experience. Then again, you’re not going to be perfect in every encounter—it’s a matter of... continuous new learning.”*

*(interview 6)*

Even though the professionals acknowledged that their education provided a solid base encountering patients well and remaining professional, they also felt that how to interact with patients in the encounter could not be learnt from books, but only by doing in practice. They admitted that this was a learning process that new employees needed to undertake to become skilled. All professions included in the study showed strong interest in continuing to attend courses and learn more.

**Balancing one’s emotions.** Not showing all their own emotions was necessary to maintain a professional approach during the encounters, to avoid burdening the patients, and to keep the focus on the subjective experience of the patient. However, keeping emotions in check was described as a balancing act. On the one hand, professionals described trying to be neutral, rather quiet, and not too eager, yet on the other, it was also important to show empathy, acceptance, and understanding to connect with the patient. Keeping a certain distance could be helpful in not getting overwhelmed by the difficult life situation of the patients. Keeping a professional distance was not mentioned exclusively by the physicians, but was generally more important to them. The distance also enabled the professionals to prepare for unexpected behaviors during the encounter. Despite knowing the patients, often for many years, professionals could not anticipate patients’ reactions and behavior in the most acute encounters. However, when the situation and the patient were calmer, the professionals could rely on their alliance with the patients.

Yet another reason to try not to show certain emotions, feelings, or thoughts was that the professionals experienced the patients as very skilled readers of facial expressions, able to catch their moods or feel their preconceptions. Despite their acute health conditions and suffering, the patients were seen as attentive to the professionals’ behavior and able to perceive fatigue, tiredness, irritation, or fear.

**Being self-insightful.** The professionals understood that they were also “just” human, and mistakes could occur during encounters with the patients. However, they were self-aware, accepted their own limitations, and reflected upon wrong assessments or misjudged situations to learn and improve as professionals. Understanding one’s own limits was seen as showing a high level of professionalism. When they were uncomfortable or feeling provoked in an encounter, they acted professionally by acknowledging those feelings and, early on, asking a colleague to take over when possible.

“A: I also think about how... certain patients are of course provoking... and they can certainly provoke me.

B: Mm-hmm.

A: So, it’s like I have a hard time with some patients’ behaviors. To be sufficiently professional, you can go to a colleague and say, ‘Can you please take over here’.

C: Mm-hmm.

A: Because it’s never helpful to continue with something when I have the feeling that this... we’re never going to get any alliance with one another.

B: Mm-hmm.

A: So that, too, I think, is part of what it means to be professional.

B: Yes.” (FG)

The professionals remained true to themselves in the encounters, while retaining a professional work role. They understood that this was necessary for them to have genuine encounters with their patients. If their own identity and professional role were too far away from each other (i.e., if they felt they had to pretend emotions or behaviors), they worried that they would not have the energy to cope with work in a long-term perspective.

“A: And I also think if I’m not being myself in the encounter, it won’t be genuine. It won’t be good for either party. It wouldn’t feel good for me if I were someone else. I don’t think I would have been able to stand it.

B: No, I don’t think so either, and it wouldn’t feel real to the patient either if I were to try to play some sort of role. No, I have to be myself—but obviously also I have to, in some way... as you say, we have to... what we’ve been saying about being professional.” (FG)

The professionals were also aware their individual personalities could not be changed and could affect the encounter. They also emphasized the importance of personal chemistry, which could help them have a good encounter and establish a better rapport. They viewed it as professional to acknowledge, allow, and accept those kinds of personal preferences.

**Using critical thinking.** Reflecting upon and re-evaluating encounters with patients and being self-critical were central elements in the professionals’ work. This demanded continuous use of their mental capacities, and the professionals could feel mentally tired from the constant mental activity required by encounters with their patients. They reflected on their own actions and thoughts, their work environment, and the patients and their everyday lives and contacts with other support services. While establishing relationships was also seen as important in somatic care, professionals in psychiatric care could often rely on only themselves and their experience, and did not have additional instruments, markers, and tools available to those assessing physical problems.

**Finding support in colleagues and managers.** Reliable colleagues and supportive managers were seen as resources for providing good encounters. The professionals expressed their need for a good work climate that fosters open dialogue across professions, teamwork and trustful relationships with colleagues, and professional (or clinical) supervision to help encounter their patients well. The professionals expressed that those needs were largely met at PES. All professions praised other professions for their competence, openness, support, and willingness to help and learn, which nurtured both inter- and intra-professional processes for mutual learning. Sometimes staff shortages, the administrative workload among nurses and physicians, and the heavy organizational structures of the healthcare system were mentioned as factors complicating encounters with the patients.

Colleagues also played an important role in handling each other’s emotions. While certain feelings were not shown to patients, they were often expressed among colleagues. Feelings of powerlessness, dejection, resignation, hopelessness, or disappointment when persons with frequent PES use did not improve despite years of personal investments from the professionals’ side were regularly experienced among the professionals.

“B: Yes... certainly I do have a sense of hopelessness sometimes. If you’ve known someone for 13 years and it never gets... it’s the same story every time... despite multiple interventions from the municipality, the county council, and various other entities, it does lead to a sense of... hopelessness, for sure. And... disappointment. Sometimes I think I’ve given so much, I give so much, and it all still goes to hell... [laughs]... for the patient.

A: Mm-hmm. Are you disappointed in the patient?

B: No... yes... maybe... The patient... of course I’ve found myself feeling disappointed in the patient, too. Yes. I have to be honest and definitely say that.” (interview 8)

Colleagues were an important source of information and second opinion and were used for guidance and support in the absence of objective measures.

### 3.3. Striving for a Meaningful Connection with the Patient

The professionals experienced the encounter as an opportunity to establish contact or connection with patients. Becoming a chameleon, hope and laughter, seeing the person, and mastering the art of interaction helped to create this connection. However, the professionals were also aware that they did not always connect with the patient and they accepted that.

**Becoming a chameleon.** The professionals learnt to adjust, to become what the patients needed them to be, and to encounter them on the appropriate level: soft and calm, direct and decisive, or physically close or more distanced. They also intuitively adjusted their body position, hand placement, their voice, and the voice pitch etc. Prior to the tuning, a quick assessment was made at the beginning of the encounter, where many factors were considered and analyzed. The professionals were primarily interested in meeting the person and his or her healthcare and human needs; however, knowing the diagnoses of the patients could be helpful, as could knowledge about the cultural and ethnic background of the patient. Also, one’s own current position in terms of prejudices, tiredness or frustration were considered, as were situational aspects, for example, the current situation of the waiting room or the time of the day. The professionals tuned their encounter based on this initial assessment and the knowledge from previous encounters. After having gained experiences in encountering the patients, the professionals could allow themselves to *feel* the right responses and trust their senses, which were safely embedded in those previous experiences.

**Working with hope and laughter.** The professionals tried to provide hope, which had a particular central role in the encounter with the patients, who often could have lost hope. The professionals understood the power of providing a sense of hope for the patients because hope had a future outlook. Another very powerful tool in the encounter was humor. The professionals showed awareness, similar to their sensitivity regarding physical contact, that humor is situation- and person-dependent. As in the case of physical contact, the professionals used their experience and knew, felt, and sensed when it was appropriate to be funny and with whom. The professionals thought that laughter could be useful in strengthening the patient–staff relationship and helped professionals and patients feel connected with each other, which was the essence of a good encounter.

**Seeing the person.** The professionals saw each encounter as individual. However, encounters with the patients, i.e., persons with frequent PES use, differed from encounters with unknown patients. With known patients, the first part of the encounter was experienced as comparatively easy, flexible, and free since the professionals were knowledgeable about the patients, their previous treatments and outcomes, and the patients’ preferences and personalities. Physical contact was one particular example that required a close alliance with the patients and was not experimented on with unfamiliar patients. The knowledge gained from previous encounters allowed for closeness and familiarity that helped the professionals connect with the patients. It also allowed other non–disease-related conversations, letting the professionals discover the patient as a whole.

*“But yes, still, the patients who show up five times a week, or 10 times a week—I’m still going to go over and greet them, take their hand, welcome them. I think it’s extremely important that we do that. That we... that we... that we see the person, regardless of whether it’s a matter of addiction or personality disturbance or psychosis or whatever—that we still see the person as a human being and support who they are.” *

*(interview 9)*

The professionals also stressed the importance of seeing the person as a fellow human being who had more than just psychiatric and healthcare needs, but also human needs, such as being confirmed and empowered. The professionals found several ways to affirm the persons with frequent PES use as fellow humans being during the encounters, with small gestures such as sitting next to instead of opposite the patient, a touch, eye contact in most cases, using the person’s name and talking about other parts of the patient’s life than disease. Being personal with the patients was thus, for many professionals, a rather common action. It was also a way to build trust and help the patient feel at ease and secure enough to open up about their lives and problems.

*“And sometimes the fact that... that it’s like... as I say, I don’t go into private matters, but I can still be personal—I don’t need to... and then I’ve understood, that goes down well.”*

*(interview 5)*

Giving room in the encounter for the patients and their stories, expectations, wishes, and needs was crucial for the professionals. The professionals also tried to confirm the patients’ decision to visit and they viewed welcoming and inviting the patient into the encounter as an investment that counteracted possible feelings of shame in patients who could still have difficulty using PES despite frequent use. The professionals showed mutuality by, for example, sitting down during the encounter instead of hovering over the patient.

**Mastering the art of interaction.** The conversation was one way of interacting to build connection with the patients, yet it was not always needed. For some patients, the professionals felt that being physically close or sharing an emotional and human connection was more important than having a conversation. There could be a meaningful and bonding experience in silence. Other patients, the professionals thought, had a strong need to conversation and be listened to. For those patients, conversations were seen as therapeutic and the professionals experienced those conversations as just as important in many cases as providing medication or other formal interventions.

To genuinely engage in a conversation, the professionals found it helpful to be present and not stressed and to allow sufficient time. Taking time showed respect and built trust. However, the time aspect was not seen as a general prerequisite to a good encounter. Connections between patients and professionals could also be established quickly or last only a few moments if there was mutual trust.

The conversation was, however, the most important part of the encounter for the professionals to diagnose and treat patients correctly. Establishing interpersonal connections built trust and made it easier to hold conversations in which patients felt ready to share their innermost thoughts, feelings, and fears. The professionals were careful not to interrupt and to let the patients decide the pace of the conversation. They tried to keep a clear, calm, and quiet tone of voice.

Conversing with the patients was experienced as an art that needed to be mastered and included skills on several spectra: from listening to talking, from being cautious to being active, from daring to ask questions and be direct to backing, coaxing and encouraging, or from steering the conversation, as several physicians described, to letting it flow loosely, as nurses did by inviting patients to open the conversation with what felt important for them. Those circular processes showed the interaction as a two-person dance requiring sensibility, responsiveness, and reciprocal skills. The professionals always tried to maintain a sense of mutuality, including mutual learning, in the conversation.

Phone conversations were experienced as more difficult than face-to-face conversations because the professionals lacked clues such as the patient’s body language and facial expressions. In such circumstances, it was thought helpful to have met the patients previously. As in a face-to-face conversation, coaxing could play an important role in building a dialogue, and attention was also paid to those things that were left out of the patient’s story. In a telephone conversation, the professionals stressed the importance of listening somewhat more carefully since there were no other cues than the patient’s story and voice.

**Being content with just an encounter.** Certain conditions such as violence, coercion, involvement of police, or patient anger could make the encounter more challenging. However, despite those obstacles, which were initially experienced as challenging and time-demanding, the professionals understood that each encounter had the potential to become good and meaningful for the patient. They thought that despite previous *uncaring* encounters or not meeting the patients’ expectations, wishes, or needs, the patients could still experience the current encounter as good and caring. However, the professionals also met patients with whom, due to lack of receptiveness or understanding from either party, they could not connect. Such encounters, however, were also characterized by respect, humility, and kindness. The professionals experienced them not necessarily as bad encounters, but simply as *just* encounters.

*“But obviously there are times when you encounter people whom you don’t... You simply don’t understand one another. You may speak the same language—Swedish—but you... somehow, you cannot meet. I don’t know if this has so much to do with the actual diagnosis. Sometimes maybe it does; but sometimes... It doesn’t always.”*

*(interview 2)*

They accept that they were not always able to make a connection with the patient instead of blaming themselves or viewing themselves as failing. They saw the encounter as a mutual process. Although they felt responsible for initiating the interaction, establishing trust, and building a non-judgmental and respectful foundation, they recognized that it required 2 parties to get involved, make a connection, and interact successfully.

The professionals stressed that only the patients could decide the quality of the encounter. Encounters professionals had forgotten could sometimes be mentioned later by patients as having been life changing, while encounters in which the professionals tried their hardest could be reported by the patients as meaningless.

## 4. Discussion

This study explored healthcare professionals’ experiences of encounters with persons who frequently use PES. The results show that the professionals experience these encounter as having both strongly caring and professional elements. These findings resonate with previous findings from other healthcare contexts, raising the importance of caring encounters and describing them with attributes such as “being there”, “uniqueness”, and “mutuality” [3]. Another study about caring encounters described the core aspects of professional caring as “being dedicated”, “being morally responsible”, “being truly present”, “being genuinely concerned”, and “being open” [13]. Those caring encounters included caring and connecting processes, developing a professional intimacy characterized by respect and compassion while maintaining a professional distance [13]. This study confirms that professionalism and caring as tightly intertwined rather than conflicting elements in encounters with persons with frequent PES use. Other research describes caring and nursing in emergency and psychiatric acute care settings as rather technical and shallow [14,22,23] and encounters with persons with mental illness as generally challenging [21]. However, in this study, professionals described providing caring interactions as meeting the person as an individual and fellow human being by becoming a chameleon and tuning in to the patients, allowing themselves to become what the patient needed them to be. Mutuality, respect, presence in the here and now, and a non-judgmental approach facilitated this caring encounter. Encountering caring healthcare professionals in acute healthcare contexts has been found to be a predictor of patient satisfaction [36]. Studies from Swedish PES found that from a professional’s perspective caring encounters were an empathetic and humane way to interact with the patient [27], while from a frequent visitor’s perspective they involved being cared for, being understood, feeling welcomed, and being treated with kindness, humanity and fairness [26]. This study is in strong agreement with those findings emphasizing the humane element of the encounter. Applying a person-centered approach in the encounter with persons who frequently use PES and paying attention to their personal stories and experiences can facilitate their recovery [37]. Hope, a positive outlook, and the concept of “power with” rather than “power over” need to be acknowledged to counter the asymmetric relationship between patients and healthcare professionals to empower persons who frequently use PES [37].

Another finding of the study is the professionals’ experience of humility and maturity in patient encounters. Despite their substantial knowledge of their patients, the professionals showed high levels of self-insight, self-awareness, and self-criticism by continuously re-evaluating and reflecting upon their preconceptions, prejudices, preferences, or difficulties that could have an impact on the upcoming interaction with the patient. This behavior is well in line with the concept of *therapeutic use of self* [12], which requires comprehensive self-understanding of one’s own feelings, values, needs, motivations, and limitations first in order to understand patients and promote their growth and health [1]. The professionals in this study acknowledged and accepted their own limitations and their fallibility as human beings. By accepting patients for who they are while also accepting themselves, the professionals highlighted the humanistic and humble aspects of the encounter. These aspects included both compassion and self-compassion; the professionals understood the importance of caring for and with the patients, but also caring for themselves. The professionals’ honest account of critically and collectively reflecting upon their limitations, mistakes, and dislikes, and their admission of occasional mental fatigue reveals and confirms their self-awareness, self-acceptance, and self-compassion [38]. Mindfulness interventions for emotionally and mentally drained mental health professionals have been shown to significantly increase self-compassion [39]. Care as a component of compassion [40] allows professionals to be mindful of their patients’ painful feelings without over-identifying and helps them to stay balanced, feel well, and have caring and professional encounters with their patients.

Finally, the results revealed the importance of colleagues, managers, and the working climate in providing caring, professional, and humane encounters. Professionals paid attention and care to their relationships not only with patients and themselves, but also with their colleagues, to create and be part of an enriching work environment. A positive, well-functioning, and satisfying workplace is a prerequisite to good encounters at PES. All interviewed professionals had common moral values and work ethics that facilitated a person-centered approach that was reflected in their encounters with patients. The importance of teamwork [41], including both smaller multidisciplinary teams and the whole organization [42], to the encounter and its interactions in psychiatric acute care is confirmed by the results of this study. Person-centered services empowering patients in their recovery processes are more likely to be facilitated when *person-centered cultures* are provided [43]. Those cultures are formed by the *care environment*, which comprises professional relationships, supportive organizational systems, and leadership, and a common set of values among the different professions [43,44]. To enable person-centered processes in encounters between healthcare professionals and patients, the same person-centered values, attitudes, and processes need to permeate the workplace’s physical, organizational and social structures [44].

Even though the infrastructure and organization of acute psychiatric care, as well as educational requirements of staff, vary across settings and contexts, the nature of PES, being an intensive, demanding, and unpredictable workplace, remains universal [5,19]. The challenges reported by healthcare professionals in acute psychiatric care from different studies are alike. They focus on increased use rates and increased workloads combined with staff shortage and limited resources, increased work stress, and unsupportive organizational cultures, loss of professional identity and unethical behaviors [19,23,45]. Thus, the clinical implications of this study for mental health nursing are particularly highlighted. To provide caring, professional, and humane encounters and interactions with persons who frequently use PES, it is necessary to recruit competent professionals who are self-aware and self-critical. Another prerequisite is that the work environment at PES is enriching, open, and supportive of professionals, including their relationships with colleagues and management. Because professionals could sometimes become mentally exhausted from constant thinking and responding in the moment, the organization should provide sufficient staffing and space for staff recovery. An important aspect of facilitating staff recovery is clinical supervision. This would not only give the professionals an opportunity to express their emotions (positive and negative) and to reflect upon themselves, their patients, and related processes or situations, but could also strengthen social structures and relationships at the workplace. PES is a particularly stressful work environment, and can be emotionally and mentally exhausting for healthcare professionals, particularly when encountering persons who frequently use PES. Routine mindfulness and self-compassion interventions could counter those processes. Finally, professionals at PES often work inter- and intra-professionally. As patients frequently encounter professionals with different professions, good communication among all involved professionals are valuable in helping both professionals and patients experience their encounters as meaningful.

To ensure trustworthiness of the findings, the authors took measures appropriate for content analysis according to Lincoln and Guba [46] as recommended by Granheim et al. [47] and followed the Consolidated criteria for reporting qualitative research (COREQ) [48]. Providing example quotes and showing parts of the analysis process helps readers judge the credibility and authenticity of the findings which could otherwise be difficult as the text always carries multiple meanings and is interpreted based on the authors’ pre-understandings. Because the authors have varying pre-understandings and interpretative ranges, to address dependability all four authors were involved in the analysis, which was finalized when they reached consensus. The readers’ ability to judge transferability was facilitated by (1) describing contexts, demographics, and professions in the introduction and method sections, and (2) emphasizing in the results and the discussion sections the positive working culture that deemed essential to good encounters and interactions between professionals and patients. *Within-method triangulation* was used to address credibility. Using individual interviews first and then conducting a focus group interview made it possible in the focus group interview to present preliminary results, which were confirmed and deepened by discussions across professions that clarified and highlighted conflicting experiences among the professionals.

## 5. Conclusions

The findings suggest that healthcare professionals at PES experience encounters with persons who frequently use PES as revealing caring, professional, and humane processes. To provide such encounters to patients, professionals need to nurture their relationships with themselves and their colleagues. Self-awareness, self-acceptance, and self-compassion were important elements in this process and required constant critical thinking and learning that could lead to mental tiredness. Each encounter was equally characterized by humility, respect, and kindness, and was highly individual. Professionals tuned in to each patient’s individuality and needs and tried to see the patient as a fellow human being. The person-centered approach in patient encounters was mirrored in the care environment at PES, which provided reliable inter- and intra-professional teamwork and supportive managers. Thus, a person-centered care environment was a prerequisite to providing caring, professional, and humane encounters. To our knowledge, this is the first study to explore healthcare professionals’ experiences of persons who frequently use PES, including faceless encounters by telephone, which were experienced as more challenging than face-to-face encounters. More knowledge is needed to provide guidance to healthcare professionals to ensure good quality encounters for patients.

## Figures and Tables

**Table 1:** Interview guide with main questions for the individual interviews.

Introduction: This interview focuses on your experiences of persons who frequently use PES. Within research, they can be defined in different ways, for example, with a minimum of 4, 5, or 6 contacts within 12 months. This study focuses on your individual experiences on who persons who frequently use PES are. Have you cared for persons who frequently use PES?
Transition questions: What are your thoughts about persons who frequently use PES and their visits? Could you describe your experiences of the encounters with them?
Main questions: How do you encounter persons who frequently use PES (visit or call in)? Can you describe examples of an encounter that you felt satisfied with/experienced as challenging? Why? In what way, if at all, do you encounter persons who frequently use PES differently from other persons? Why do you think that is? In what way, if at all, do you adjust your encounter with them? Why do you think that is? How do you communicate with persons who frequently use PES? How do you create a trustworthy and safe environment for these persons? What emotions does the encounter with persons who frequently use PES trigger in you? Can you describe an example of an encounter that triggered positive/negative emotions? How do you handle those emotions?
Closing questions: Is there anything I have not asked that you would like to add? Can I get back to you if I have any further questions? Would you be interested in participating in a focus group interview? Summary

**Table 2:** Summary of the results of the analysis process in form of sub-themes and themes revealing the professionals’ experiences of the encounter with the patients.

Sub-Themes	Themes
Allowing for constant learning from experience Balancing one’s emotions Being self-insightful	Nurturing the encounter with oneself and colleagues for continuous, professional improvement
Using critical thinking Finding support in colleagues and managers	
Becoming a chameleon	Striving for a meaningful connection with the patient
Working with hope and laughter	
Seeing the person	
Mastering the art of interaction Being content with just an encounter	

## Appendix A

**Table A1:** Participants.

	Individual Interviews (*n* = 19)	Focus Group (*n* = 1)
Gender		
Men	6	3
Women	13	3
Age m (range)	47 (29–70)	48 (30–69)
Professions		
Assistant Nurse	3 (13–17) *	1
Registered Nurse	10 (1–40) *	4
Intern Physician	2 (−) *	0
Resident Physician	4 (1–4) *	1
Country of Birth		
Sweden	15	6
Other	4	0

* Range in years of professionals’ experience of working at a PES.

## Appendix B

**Table A2:** Examples of the analysis process and development of the sub-themes and themes.

Example of Meaning Unit	Condensed Meaning Unit-Description	Condensed Meaning Unit-Interpretation	Sub-Theme	Theme
No doubt it’s... experience is part of it, plus... Yes, I think it has to do with experience too—that you’ve experienced similar situations before, that you recognize certain patterns... and so on—but not always. Sometimes it doesn’t help. (interview 13)	Experience is part of it: you’ve experienced similar situations and you recognize certain patterns. Sometimes it helps, sometimes not.	Finding patterns in encounters	Allowing for constant learning from experiences	Nurturing the encounter with oneself and colleagues for continuous, professional improvement
I try not to show (my frustration). Then of course it sometimes happens that you... but I actually try not to do that. I hope not, because... it’s like I said before, that they can tell if you... They notice things very, very well... (interview 3)	I try not to show (my frustration). They can tell, they notice a lot of things.	Keeping emotions in check	Balancing one’s emotions	
Probably the first thing is just to be able to acknowledge to yourself that this is actually not going to work—like ‘Maybe I’m really having trouble connecting with this person’. Or ‘It’s triggering something in me that’s making me kind of uncomfortable’. (interview 6)	The first thing is to acknowledge to yourself that this is actually not going to work—‘I’m having trouble connecting with this person’.	Seeing one’s own limitations	Being self-insightful	
*A: ... because, well, this job requires a lot of energy*—*mental energyy*—*because every person who calls wants something from me*—*emotionally, usually, of course.* *B. Mm-hmm.* *A: And then, obviously, you get kind of drained, you know. Everyone has their bucket... how should I put it?...their bucket of energy—how much involvement you can stand.* *B: Mm-hmm.* *A: But that’s no doubt something you just have to learn, I think—otherwise you’re likely to... you’ll get too involved and you won’t have the energy for it.* *C: Mm-hmm.* *A: Then again, we have each other to go and vent to.* *B: That’s… that’s an important part afterwards… (FG)*	Patient encounters take a lot of energy and leave you drained. Each person has to learn how far they can go in getting involved, how much they have the energy for. We do have one another to vent to afterwards—that’s important.	Reflecting on work and encounters Acknowledging the importance of colleagues	Using critical thinking Finding support in colleagues and managers	

## References

[1] Peplau H.E. (1988). Interpersonal Relations in Nursing: A Conceptual Frame of Reference for Psychodynamic Nursing.

[2] Holopainen G., Kasén A., Nyström L. (2014). The space of togetherness—A caring encounter. Scand. J. Caring Sci..

[3] Holopainen G., Nyström L., Kasén A. (2019). The caring encounter in nursing. Nurs. Ethics.

[4] Fleischer S., Berg A., Zimmermann M., Wüste K., Behrens J. (2009). Nurse-patient interaction and communication: A systematic literature review. J. Public Health.

[5] Schmidt M. (2018). Frequent visitors at the psychiatric emergency room—A literature review. Psychiatr. Q..

[6] Snellman I., Gustafsson C., Gustafsson L.K. (2012). Patients’ and caregivers’ attributes in a meaningful care encounter: Similarities and notable differences. ISRN Nurs..

[7] De Leeuw M., van Meijel B., Grypdonck M., Kroon H. (2012). The quality of the working alliance between chronic psychiatric patients and their case managers: Process and outcomes. J. Psychiatr. Ment. Health Nurs..

[8] King B.M., Linette D., Donohue-Smith M., Wolf Z.R. (2019). Relationship Between Perceived Nurse Caring and Patient Satisfaction in Patients in a Psychiatric Acute Care Setting. J. Psychosoc. Nurs. Ment. Health Serv..

[9] Delmar C. (2012). The excesses of care: A matter of understanding the asymmetry of power. Nurs. Philos..

[10] Priebe S., Mccabe R. (2008). Therapeutic relationships in psychiatry: The basis of therapy or therapy in itself?. Int. Rev. Psychiatry.

[11] Paterson J., Zderad L. (1976). Humanistic Nursing.

[12] Travelbee J. (1971). Interpersonal Aspects of Nursing.

[13] Halldorsdottir S. (1996). Caring and Uncaring Encounters in Nursing and Health Care: Developing a Theory. Ph.D. Thesis.

[14] Nyström M., Dahlberg K., Carlsson G. (2003). Non-caring encounters at an emergency care unit–a life-world hermeneutic analysis of an efficiency-driven organization. Int. J. Nurs. Stud..

[15] Gustafsson L.K., Snellma I., Gustafsson C. (2013). The meaningful encounter: Patient and next-of-kin stories about their experience of meaningful encounters in health-care. Nurs. Inq..

[16] Ford J.S. (1990). Caring encounters. Scand. J. Caring Sci..

[17] Cleary M., Horsfall J., O’Hara-Aarons M., Jackson D., Hunt G.E. (2012). Mental health nurses’ perceptions of good work in an acute setting. Int. J. Ment. Health Nurs..

[18] Wise-Harris D., Pauly D., Kahan D., de Bibiana J.T., Hwang S.W., Stergiopoulos V. (2017). “Hospital was the only option”: Experiences of frequent emergency department users in mental health. Adm. Policy Ment. Health.

[19] Buus N. (2011). Categorizing “Frequent Visitors” in the Psychiatric Emergency Room: A Semistructured Interview Study. Arch. Psychiatr. Nurs..

[20] Koekkoek B., van Meijel B., Hutschemaekers G. (2006). “Difficult patients” in mental health care: A review. Psychiatr. Serv..

[21] Marynowski-Traczyk D., Broadbent M. (2011). What are the experiences of emergency department nurses in caring for clients with a mental illness in the emergency department?. Aust. Emerg. Nurs. J..

[22] Waldemar A.K., Esbensen B.A., Korsbek L., Petersen L., Arnfred S. (2019). Recovery-oriented practice: Participant observations of the interactions between patients and health professionals in mental health inpatient settings. Int. J. Ment. Health Nurs..

[23] Wyder M., Ehrlich C., Crompton D., McArthur L., Delaforce C., Dziopa F., Powell E. (2017). Nurses experiences of delivering care in acute inpatient mental health settings: A narrative synthesis of the literature. Int. J. Ment. Health Nurs..

[24] Harris B., Beurmann R., Fagien S., Shattell M.M. (2016). Patients’ experiences of psychiatric care in emergency departments: A secondary analysis. Int. Emerg. Nurs..

[25] Shattell M.M., Andes M., Thomas S.P. (2008). How patients and nurses experience the acute care psychiatric environment. Nurs. Inq..

[26] Schmidt M., Ekstrand J., Bengtsson Tops A. (2018). Self-reported needs for care, support and treatment in persons who frequently visit psychiatric emergency rooms in Sweden. Issues Ment. Health Nurs..

[27] Schmidt M., Pernilla G., Stjernswärd S., Ann-Christin J. (2020). Professionals’ perspective on needs of persons who frequently use psychiatric emergency serivces. Issues Ment. Health Nurs..

[28] The Swedish Parliament (Svenska riksdagen) (2017). [Health and Medical Care Act]. HSL Hälso- Och Sjukvårdslag, 2017:30.

[29] The National Board of Health and Welfare (Socialstyrelsen) (2009). [National Indicatiors for Good Care]. Nationella Indikatorer för God Vård.

[30] The National Board of Health and Welfare (Socialstyrelsen) (2015). [To Encounter in Healthcare]. Att Mötas i Hälso- Och Sjukvård.

[31] Thurmond V. (2001). The point of triangulation. J. Nurs. Scholarsh..

[32] Polit D.F., Beck C.T. (2016). Nursing Research: Generating and Assessing Evidence for Nursing Practice.

[33] Graneheim U.H., Lundman B. (2004). Qualitative content analysis in nursing research: Concepts, procedures and measures to achieve trustworthiness. Nurse Educ. Today.

[34] Krippendorff K. (2013). Content Analysis: An Introduction to Its Methodology.

[35] World Medical Association (WMA) Declaration of Helsinki: Ethical principles for medical reserarch involving human subjects. Proceedings of the 64th WMA General Assembly.

[36] Welch S.J. (2010). Twenty years of patient satisfaction research applied to the emergency department: A qualitative review. Am. J. Med. Qual..

[37] Barker P., Buchanan-Barker P. (2010). The tidal model of mental health recovery and reclamation: Application in acute care settings. Issues Ment. Health Nurs..

[38] Raab K. (2014). Mindfulness, self-compassion, and empathy among health care professionals: A review of the literature. J. Health Care Chaplain..

[39] Raab K., Sogge K., Parker N., Flament M.F. (2015). Mindfulness-based stress reduction and self-compassion among mental healthcare professionals: A pilot study. Ment. Health Relig. Cult..

[40] Wollenburg K.G. (2004). Leadership with conscience, compassion, and commitment. Am. J. Health Syst. Pharm..

[41] Cleary M., Hunt G.E., Horsfall J., Deacon M. (2012). Nurse-patient interaction in acute adult inpatient mental health units: A review and synthesis of qualitative studies. Issues Ment. Health Nurs..

[42] McAllister S., McCrae N. (2017). The therapeutic role of mental health nurses in psychiatric intensive care: A mixed-methods investigation in an inner-city mental health service. J. Psychiatr. Ment. Health.

[43] McCormack B., Dewing J., Breslin L., Coyne-Nevin A., Kennedy K., Manning M., Peelo-Kilroe L., Tobin C., Slater P. (2010). Developing person-centred practice: Nursing outcomes arising from changes to the care environment in residential settings for older people. Int. J. Older People Nurs..

[44] Wolf A., Ekman I., Dellenborg L. (2012). Everyday practices at the medical ward: A 16-month ethnographic field study. BMC Health Serv. Res..

[45] Zarea K., Malek F.-M., Shahram B., Tahery N. (2018). Challenges encountered by nurses working in acute psychiatric wards: A qualitative study in Iran. Issues Ment. Health Nurs..

[46] Lincoln Y.S., Guba E.G. (1985). Naturalistic Inquiry.

[47] Graneheim U.H., Lindgren B.M., Lundman B. (2017). Methodological challenges in qualitative content analysis: A discussion paper. Nurse Educ. Today.

[48] Tong A., Sainsbury P., Craig J. (2007). Consolidated criteria for reporting qualitative research (COREQ): A 32-item checklist for interviews and focus groups. Int. J. Qual. Health C.

